# The common sense model applied to hepatitis C: a qualitative analysis of the impact of disease comparison and witnessed death on hepatitis C illness perception

**DOI:** 10.1186/s12954-015-0054-1

**Published:** 2015-06-20

**Authors:** Stella A. Safo, Abigail Batchelder, Deena Peyser, Alain H. Litwin

**Affiliations:** Department of General Internal Medicine, Albert Einstein College of Medicine, 111 East 210 St., Bronx, NY 10467 USA; University of California, San Francisco, CA USA; Rutgers University, Newark, NJ USA

**Keywords:** Hepatitis C (HCV), HIV/AIDS, Illness perception, Treatment barriers, People who inject drugs (PWID), Substance use

## Abstract

**Background:**

Hepatitis C virus (HCV) accounts for 15,000 deaths in the United States yearly because people living with HCV are not identified in time to seek treatment, are ineligible for or refuse treatment, or face structural impediments to obtaining treatment such as lack of access to health care or lack of insurance. People who inject drugs (PWID) comprise a large proportion—estimates of up to 60–70 %—of current and new HCV infected individuals and face many barriers to completing HCV treatment.

**Methods:**

We conducted 30 qualitative semi-structured interviews of current and former PWID seeking HCV treatment at an opioid-agonist treatment facility in New York City. We used thematic analysis, informed by grounded theory, to examine perceptions of HCV and decisions to initiate HCV treatment. We analyzed the themes that emerged via the common sense model (CSM) of illness perception theoretical framework.

**Results:**

Using thematic analyses, two major themes emerged related to engagement in HCV treatment. First, participants independently compared HCV to HIV, and in so doing, emphasized the potential fatality of HCV and the need for treatment. Second, participants described witnessing others suffer or die from untreated HCV and expressed how these recollections impacted their desire to undergo treatment themselves. Together, these themes contributed to the way participants perceived HCV and informed their decisions to initiate treatment. Both themes reflect the CSM’s “self-regulation” process, which posits that understanding the causes and consequences of an illness impacts one’s ability to seek treatment to overcome this illness state.

**Conclusions:**

This paper offers insight into how clinicians can better understand and utilize HCV illness perceptions to evaluate willingness to engage in HCV treatment among PWID considering antiviral treatment modalities.

## Background

The burden of disease from hepatitis C virus (HCV) in the United States remains high, with estimates of approximately three million infected people and peak rates predicted for the 2020s–2030s [1]. The development of novel HCV direct acting antivirals (DAAs)—which improve the tolerability and convenience of HCV treatment [2, 3]—is changing the HCV treatment landscape [4]. Individuals who could not tolerate HCV treatment in the past are successfully undergoing treatment with new DAAs to achieve sustained virologic response (SVR) [3]. However, among the roughly three million individuals infected with HCV in the US, only 43 % are in medical care and only 9 % have achieved SVR [5]. In the context of this low treatment uptake, the importance of addressing barriers to engagement in care among individuals living with HCV remains a vital public health effort in HCV control.

People who inject drugs (PWID) comprise approximately 60–70 % of new HCV infections in the US [1] and are particularly vulnerable to HCV infection for various reasons, including needle sharing and high-risk sexual behavior [6–8]. PWID have historically faced structural, systematic, clinical, and individual-level barriers to engaging in and completing HCV treatment [9–12], and much research has investigated factors that impede this group from undergoing HCV treatment [4, 13, 14]. Some inhibiting factors include social stigmatization, negative encounters with the medical field, provider reluctance to prescribe HCV-medications to PWID, lack of availability of effective new DAAs, and structural barriers such as lack of stable housing, fear of legal action, and lack of insurance [4, 12–17]. Once PWID living with HCV do engage in care, some may struggle to complete treatment [14]. Historically, this lack of treatment initiation was partially due to poor tolerability of treatment options, consisting primarily of pegylated interferon alfa-2a and ribavirin, that were lengthy and have adverse side effects [18, 19]. In countries where DAAs are available and approved, such as the US [3, 4, 8], their uptake continues to be affected by PWID’s reluctance to and difficulty in engaging in HCV care [20–22]. In light of these challenges, PWID remain an important yet difficult to engage group, whose successful treatment could have a significant influence on HCV morbidity and mortality [1, 15].

The extensive body of work on ways in which people with HCV conceptualize HCV suggests that an individual’s understanding of his/her illness affects his/her willingness to get screened for HCV, to consider initiating treatment, and to continue with treatment once started [4, 20, 23, 24]. Studies using mixed-methods to examine perceptions surrounding HCV treatment uptake in PWID have found that motivators for seeking treatment include, but are not limited to, a desire to return to a previously healthy state, efforts to avoid spreading HCV to others, and a desire to avoid the fear and stigma associated with HCV [4, 12, 13, 17, 24–26]. These studies reinforce that PWID’s illness perceptions of HCV play a role, among many other factors, in treatment initiation.

The common sense model (CSM)—or illness perception model—theorizes that individuals create abstract representations of their illnesses based on various factors (such as symptoms or acquired knowledge) to help to explain their illnesses to themselves and to manage their disease states [27, 28]. These illness perceptions are based on cognitive and emotional experiences of the threat of the illness, on experienced symptoms, and on knowledge about causes and consequences of illness [29]. The process of “self-regulation” is foundational to the CSM: individuals engage in a “self-regulation” process that includes practicing behaviors that will return them to their perceived normal state of health. According to the CSM, the process of regaining normal health starts with the belief that one is afflicted by an illness and that this illness requires some change in behavior to return to the pre-illness baseline. Thus, the CSM model posits that an individual’s illness perception is the primary impetus that drives him or her to engage in disease modifying behaviors such as engaging in treatment and adhering to medication regimens [30–32]. The CSM has been applied to many chronic diseases, including diabetes, hypertension, and asthma [30, 32, 33].

While studies report physical symptoms associated with HCV [19, 26, 34], the lack of clear and definable symptom presentation may make HCV difficult for individuals to recognize [1, 17], which may impact their motivation to engage in treatment. Therefore, other sources of information contribute to the way people living with HCV perceive their illness and feel motivated to seek out and engage in treatment. Potentially because of the shared risk factors, several studies have compared HCV to HIV. Studies using HIV as a benchmark to understand HCV predominantly find that PWID view HIV as more deadly than HCV [4, 11, 19, 20, 22–24, 35, 36]. Additionally, potentially as a result of the lack of clear symptom presentation [17], the impact of witnessing the effect of HCV in others has been noted as a meaningful experience among people living with HCV. Witnessing others suffer from HCV has been shown to both encourage and discourage individuals from seeking treatment [4, 11, 23].

In this study, we sought to elucidate participants’ HCV illness perceptions among predominantly Hispanic and African American substance users who had undergone treatment within an integrated HCV treatment program. This study builds on existing literature by using qualitative analysis to evaluate the illness perceptions around HCV within a longstanding opioid-agonist treatment program that maintains a nonjudgmental culture and peer involvement in an urban environment. We use exemplary quotes to highlight the themes of HIV as a benchmark for comparison and the impact of witnessing death and suffering in others as two topics that demonstrate the CSM at work within illness constructions around HCV.

## Methods

### Setting

This study is a qualitative sub-study of a randomized controlled trial (RCT) examining HCV treatment among people enrolled in an opioid-agonist treatment program who are current and former injection drug users. The RCT was conducted between 2008 and 2013 at two primary addiction care clinics in the Division of Substance Abuse at Albert Einstein College of Medicine in the Bronx, New York. It aimed to evaluate the efficacy of HCV treatment administered as directly observed therapy compared to self-administered therapy. Treatment was provided as either pegylated interferon alfa-2a and ribavirin or as telaprevir in combination with pegylated interferon alfa-2a and ribavirin. The specifics of the parent RCT trial are described elsewhere [37].

In the qualitative sub-study that provided data for this paper, providers informed eligible participants about this study and study staff called individuals who completed HCV treatment. All individuals reached by phone agreed to participate, one of whom did not attend two scheduled interviews. Study staff did not provide HCV or HIV education. The full multidisciplinary HCV treatment team included internal medicine physicians, physician assistants, nurses, substance abuse counselors, and a psychiatrist. In addition, HCV support groups and a formal peer educator program were offered at the clinic sites [38, 39]. Basic education on HCV (transmission, treatment, etc.) was provided at entrance to the on-site HCV program, which was remote (>6 months) to the administration of the semi-structured interview. The educational curriculum did not formally describe HCV within the context of HIV.

### Participants

We interviewed 30 current or former participants in the HCV treatment program (Table 1). All participants had a history of illicit substance use and were seeking drug treatment at an opioid-agonist program. Active drug use was not a contraindication to treatment. Approximately 52 % of patients in the program used illicit substances in the 6 months prior to treatment. All participants were English speakers and ten were women. Twenty-two participants were self-identified as Hispanic, four as African American, and four as others. All participants were enrolled in Medicaid. Seven participants were co-infected with HIV and HCV. All participants received directly administered weekly pegylated interferon injection. Twenty-two participants were on regimens of pegylated interferon alfa-2a plus ribavirin only and eight participants also received telaprevir in combination with pegylated interferon alfa-2a/ribavirin. Twenty participants achieved SVR and ten did not achieve SVR. Twenty-five participants completed HCV treatment, and five discontinued antiviral treatment early.

**Table 1:** Demographic characteristics, *N* = 30

	Participants, *N* (%)
Age (years)^a^	52 (46–59)
Race	
Hispanic	22 (73)
African American	4 (13)
Others	4 (13)
HIV-HCV co-infection	7 (23)
HCV treatment^b^	22 (73)

^a^Given as median (IQR)

^b^Treatment administered as pegylated IFN and ribavirin; eight separate participants received pegylated IFN, telaprevir, and ribavirin

### Data source and collection

Qualitative interviewer and co-author (AB) conducted 45–90-min-long one-on-one interviews with the participants at the opioid-agonist clinics. We used a semi-structured interview guide that explored participants’ perceptions of HCV, experiences initiating and undergoing HCV treatment, HCV medication adherence, and substance use. The Institutional Review Boards of the Albert Einstein College of Medicine approved this study.

### Analysis

Our thematic analytic approach followed Braun’s six steps of qualitative analysis and included elements of grounded theory [40, 41]. Initially, one co-author (AB) listened to and read all interviews, open coding and noting memos on how themes seemed to relate to one another in the interviews. Open codes, memos, and parts of transcripts were brought to the full research team multiple times to discuss concepts and categories as well as relationships. This process resulted in a working coding tree that was iteratively refined by obtaining feedback on transcripts, codes, concepts, and categories. This process also resulted in several visual models, depicting how the codes related to one another in the interviews. Subsequently, the transcripts were reviewed by two co-authors (AB and DP), which resulted in refinement of the coding tree and theoretical conceptualization. All discrepancies were brought to the full research team, and the tree was iteratively refined resulting in the final coding structure. Each transcript was then double coded using the final coding structure by at least two co-authors (AB, DP, or SS) blind to each other’s codes. Final coding discrepancies were discussed by co-authors until a decision was reached. We coded all interviews using NVIVO Version 10.

The themes described in this paper emerged as key factors relating to participants’ HCV illness perceptions and reasons for initiating and engaging in HCV treatment. Of note, while numerous topics emerged throughout the aforementioned process, we focus on two themes in this paper that were described as meaningfully associated with engagement in HCV care: comparison of HIV to HCV and the impact of witnessing the effect of HCV in others. These themes were consistent with the CSM of illness perception; therefore, the results are presented within that existing theoretical framework.

## Results

Through our analyses, two components emerged as primary factors contributing to participants’ conceptualization of HCV and seemed related to engagement in HCV treatment. First, all participants spontaneously compared HCV to HIV and, in so doing, used HIV as a benchmark to better understand HCV infection. Subthemes for HIV as a benchmark were HCV as being worse than, the same, or better than HIV. Second, participants explored how the experience of witnessing untreated HCV in others affected their conceptions of HCV illness. Subthemes of witnessing HCV-related suffering were the impact of witnessing in loved-ones and in acquaintances and the impact of witnessing in encouraging engagement in treatment.

### Component I: HIV as a benchmark for understanding HCV

Many participants described conceptualizing HCV in comparison to HIV. They described being unfamiliar with HCV while being knowledgeable about HIV. Some participants conceptualized HCV as being less severe than HIV, some noted HCV to be similar to HIV in severity, and a few considered HCV to be more severe than HIV. Regardless of perceived severity, participants compared HCV to HIV to better understand the former and, in so doing, depict the application of the CSM.

#### HCV as less severe than HIV

Those participants who considered HIV to be more severe than HCV emphasized the fatality and incurability of HIV. One participant succinctly stated, “HIV is like a death sentence”. Another participant emphasized the fatality of HIV but instead focused on the lack of curability:

“HIV doesn’t have a treatment—you’re not going to get well. [Doctors] can tame it. They can treat it, but you’re not going to get rid of it.”

Another participant noted HCV was “not as serious as HIV,” but that it is “somewhat” serious “because it’s something that is quiet in your system.” In contrast to HIV’s perceived permanence, some participants emphasized the curability of HCV as a comparison to the incurability of HIV. For example, this participant explained:

“…If I was told that I had Hep C [HCV], I would say to myself, at least I don’t have HIV. I’ve seen [that HCV] wasn’t a real threatening disease. I seen it like a few pills or whatever and everything is okay.”

#### HCV as same as HIV

In contrast to the perception of HIV as more severe than HCV, some participants viewed HCV and HIV as being equally deadly, particularly if left untreated. For example, after describing how AIDS reminded him of HCV, this participant explained:

“They’re [HIV and HCV] all bad, and, I think Hep C should be looked at just like if you had AIDS…It could do damage to you, it could kill you.”

Another individual noted the similarity in HIV and HCV but focused instead on the fatality of HCV over time:

“I view Hep C like HIV, because after the long run, [HCV] is fatal…you could die off of this. I don’t see it like a broken arm or a cut in your finger or something.”

Finally, one participant noted that the similarity of HIV to HCV is only in the context of individuals not undergoing HCV treatment, which he explains as “not taking it seriously:”

“They’re [HIV and HCV] the same. If not dealt with properly they can both lead to the same place. They both have to be taken seriously. [HCV] has to be taken seriously and not judged by how much pain I have.”

#### HCV as more severe than HIV

A few individuals viewed HCV as worse than HIV. For these people, the higher mortality rates of HCV made a lasting impression on them. For example, when asked to elaborate on his initial response in which he stated that HCV is worse than HIV, this participant explained:

“I think [HCV] is worse [than HIV] because there’s more people infected [with HCV] than people with HIV. Four times as many. There’s only a million people infected with HIV, but there’s four million infected with Hep C. And they do call [HCV] the silent killer.”

Participants also described the fast-acting nature of HCV. One participant spoke of knowing an individual who was co-infected with HIV and HCV; while this loved-one had untreated HIV for years, once he was diagnosed with liver failure caused by HCV, he died very quickly.

Another participant expounded on the fatality of HCV infection, especially in the context of high-risk behaviors:

“I think [HCV’s] even worse than HIV now … It’s killing more people than HIV and quicker, especially if you drink. And a lot of people do drink and they’re still drugging so it’s going to kill them faster because they’ve been using and they’re still in the streets. They got HIV. But they haven’t died of the HIV… they die of [HCV].”

### Component 2: witnessing HCV-related suffering and death in others

In addition to using HIV as a construct to form HCV illness perception, the experiences of HCV-infected family and acquaintances also impacted participants’ illness perceptions. Participants spoke of the impact of illness in kin, of the impact of illness in acquaintances, and of the lasting impact of these recollections on their willingness to engage in HCV treatment.

#### Impact of illness in kin

Descriptions of the impact of HCV in family members were often very detailed and made a lasting impact on participants. One participant recalled witnessing his brother die of HCV-related liver disease. The following account describes the participant’s highly detailed memory:

“My brother died of Hep C and then his wife died of Hep C… that’s a very ugly death—the color, the way they swell. They lose their leg movements completely, some arm movements. My brother was never crippled, but he was in a wheelchair. He couldn’t walk, he couldn’t even wipe his own butt. It got real bad…I couldn’t hold a conversation with him because he’s being fed by tubes, a machine is breathing for him and he’s swelling up. He always weighed like 160 lbs, he’s solid, the Marine type. [Once he became ill] he swelled up like three times his size and then he got black, he had the jaundice thing. I just couldn’t bear to see him so I told the doctors to pull the plug, just let him die. It was a hard decision…”

Years later, this participant is able to recall particular details of his brother’s hospitalization and death, recounting his brother’s ill appearance and the medical equipment used near the time of his death. His brother died an “ugly death,” a description that other participants echoed with similarly illustrative examples of seeing death and suffering in loved-ones.

Some participants did not know at the time that their loved-ones were dying of liver disease and realized the true cause of death only after contracting HCV themselves. For example, this participant described his childhood memory of a sick parent:

“[My mother] had cirrhosis of the liver… she was very small, and her stomach was so big, and we said to the [neighborhood] kids she was having a baby. And one day, one kid said, you said [that she was pregnant] last year, and the year before that. …I didn’t connect that with [HCV]. But, as I started going to the groups, and I started taking notes, and learned that my liver could get to where she was at the last stop.”

This example exemplifies participants’ vivid recollections of witnessing loved-ones struggle with HCV, recollections that haunted this participant years later. Furthermore, this participant’s verbalization of how he feared he could die just as his mother died was echoed by other individuals in our study about their loved-ones.

#### Impact of illness in acquaintances

In addition to family members, some participants were moved by the experience of seeing non-family members suffer from HCV. For example, this participant’s example of witnessing HCV-related death is stated in more general terms, with a reference to “people” rather than to someone close to him:

“You know, I seen *people* that died with Hep C and it’s not pretty. It’s a situation that you suffer after a while—it starts eating you up—your cells and everything…” [Italics mine].

Though the individual did not specify who the “people” were, his statement indicated that the destructive physical outcomes of HCV left a lasting impression on him. Similarly, another participant referred to a number of unnamed acquaintances living with HCV:

“I feel like [HCV’s] a thing that really kills you. I see a lot of *people* die of it now. Now I open my eyes to it and say ‘wait a minute.’ Thank God I [started treatment] now, before it got worse since I see so many *people* dying of it” [Italics mine].

These accounts suggest that regardless of the degree of familial or social closeness, witnessing suffering impacts individuals’ perception of HCV.

#### Impact of witnessing on treatment uptake

Participants described the lasting impact of witnessing HCV-related deaths. Some participants explicitly stated how the proximity to HCV’s fatal outcomes encouraged treatment initiation. For example, one participant related the effect that his aunt’s experience with HCV had on his decision to begin treatment:

“I really thought about it because when [my doctor] told me about all the side effects I was like, ‘I don’t want to feel like that.’ And then again, I thought about my [deceased] aunt and I said, ‘if I can’t make it through the whole six months, at least I’ll try.’ That’s what kept me going, thinking about getting rid of that thing. Because, [HCV] could kill you.”

Similar to this participant whose aunt’s death inspired him to overcome his fears of treatment, other participants emphasized how remembering loved-ones living with HCV encouraged them to continue treatment despite adverse medication side effects. As this participant explained:

“To me [taking medications] is like a obligation. I do this regardless of how I feel about the injections or the medication or whatever. It’s something that I have to do because…I knew several individuals that did have Hep C and they’re no longer with us. They have died from it. And I don’t want to die.”

## Discussion

This study showed that among PWID enrolled in a comprehensive multidisciplinary HCV treatment program within an addiction care center, participants’ HCV illness perceptions were substantially influenced by comparisons to HIV and by witnessing others’ suffer from HCV. Among this population of ethnic minority PWID, these components guided participants’ understanding of their illness states and contributed to the initiation of and perseverance through HCV treatment. Both thematic findings in our study support the theoretical framework laid out by the CSM. The CSM posits that “self-regulation”—the process of returning to a previously health state—is informed by one’s understanding of the causes and consequences of illness. This understanding encourages individuals to take the steps necessary to return to a healthy state [28, 31]. In our study, we found that participants used perceptions of HIV and witnessing suffering in others to better to understand the causes and consequences of HCV infection. Our participants suggest that illness perception may have led to engaging in healthy behaviors such as treatment uptake. With witnessing, we have clear examples of individuals explicitly stating how seeing others suffer or die from HCV motivated them to engage in treatment, an example of the CSM’s focus on consequences at work. Likewise, in keeping with the CSM’s focus on the need to understand the cause of the disease, we found that our participants spoke of HCV within the context of HIV to better understand HCV severity. With both themes, we see how the application of the CSM to HCV illness perceptions broadens participants’ understanding of HCV illness and facilitates engagement in treatment. This real-world application of the CSM suggests that future qualitative studies of illness perceptions in PWID could be better understood using this framework.

Furthermore, our study found diverse perceptions of severity of HCV as compared to HIV. Previous studies noted that PWID with HCV often evoked HIV as a disease for comparison [19, 20, 23, 24, 35]. However, in these studies, HIV is described as worse than HCV or as something one should avoid at all costs. For example, Davis et al. found that PWID often practiced “safe” drug use behavior out of a fear of contracting HIV rather than HCV [23]. Likewise, other qualitative analyses highlighted that among PWID, HIV was seen as more severe than HCV [20, 24, 35]. The tendency to view HIV as more severe than HCV may have been partially due to greater familiarity with HIV as it has been a disease in the public consciousness since the early 1980s (compared to HCV’s relatively recent emergence) and because of the similar risk factors between HCV and HIV (i.e., both are viral infections transmitted by sharing injection needles or from unsafe sex). Conversely, the current tendency to view HCV as severe as or as more severe than HIV may reflect the timing of our study relative to previous studies because since 2007, HCV has led to higher mortality than HIV in the United States [42]. Contrary to previous literature, our study reveals a more assorted view of HCV where HCV is seen as the same, better, or worse than HIV. While this comparison to HIV is made in varying degrees of severity, participants’ tendency to continue to use HIV as a benchmark to understand HCV has clinical implications. Since studies have found that participants’ knowledge of HCV may often be misinformed [20, 24, 26], using HIV as a reference point could offer clinicians one mechanism to address and counter the incorrect beliefs PWID with HCV may hold about HCV.

Consistent with previous published literature, we found that the process of witnessing suffering and death in others with HCV often greatly influenced participants’ illness perception by contributing to their understanding of the consequences of their own infected state [4, 19]. Many of our participants felt motivated to engage in treatment after hearing about or witnessing the demise of individuals who failed to seek treatment. Once initiated, some participants’ recollections of loved-ones’ health struggles helped them to continue and complete treatment despite concerns of experiencing adverse medication side effects. Previous studies have found that witnessing has a dual impact. Some studies have shown that witnessing HCV-infected loved-ones undergo such procedures as liver biopsies deterred individuals from wanting to engage in HCV care [11, 17, 19]. In contrast, others such as Sublette’s study of PWID infected with HCV in West Sydney found that participants cited loved-ones who had been screened or treated for HCV as their reasons for engaging in medical care [4, 22]. Consistent with these latter studies, participants in our study were more likely to be positively influenced by the experience of others to engage in treatment, a finding made unique by our predominantly minority population receiving care at an opioid-agonist treatment program. Moreover, we found that not only loved-ones such as kin or close friends influenced participants’ views of HCV, but also acquaintances and general “people,” suggesting that the sphere of influence of witnessing extends beyond one’s immediate circle.

This study has several limitations. Because a number of individuals were co-infected with HIV, the use of HIV as a construct for comparison may have been higher than in another group with less co-infection. We note, however, that the comparisons of HIV to HCV occurred even in those who were not co-infected. This finding is consistent with a recent study by Chen et al. on perceptions of HCV among HIV mono- and co-infected individuals, which found that both groups shared similar knowledge and attitudes towards HCV [43]. Another limitation is that our study was based on an urban sample of individuals enrolled in an opioid-agonist treatment program. It is unclear if similar views would be conveyed in areas differing from this setting (e.g., non opioid-agonist, non-urban). Finally, our study interviewed people who had already completed HCV treatment, which could have impacted how much participants focused on certain topics such as medication side effects or importance of cure.

The results of this study have several clinical implications. By understanding the role illness perceptions may play as a driver of treatment uptake, HCV providers may choose to spend some time exploring disease representations in this group both before and during HCV treatment. Because most people living with HCV likely have clear illness perceptions of HIV, providers can explore how conceptualizations of HIV compare and contrast with individuals’ HCV illness perception. From this starting point, providers may reinforce accurate illness perceptions and correct inaccurate ones. Providers can also explore whether patients have witnessed the death of anyone with HCV and can evaluate how this experience shaped his/her illness perception. Providers may then use direct discussion, support groups, or peer educators to help modify illness perceptions based on witnessed death or suffering. Specifically, we note that while clinical providers such as medical doctors or nurse practitioners are often at the forefront of delivering HCV treatment, we believe that counseling around engaging in HCV treatment can be done via peer-led support groups, peers, and hepatitis educators as well.

## Conclusions

Our study demonstrates that PWID with HCV formed numerous views of HCV illness that were consistent with the common sense model. Specifically, participants’ HCV illness perceptions were founded on using HIV as a benchmark to understand HCV and on the impact of witnessing HCV-related suffering in others. These illness perceptions are important because they may inform how providers engage PWID in HCV treatment. The emergence of novel and effective DAAs with short treatment courses greatly expands the potential to successfully cure individuals with HCV. Within this landscape of improved treatment options, people living with HCV should be urgently identified and engaged in care. We suggest that a focus on illness perception may be one tool, among many, in the arsenal to successfully engage PWID living with HCV in care.

## Abbreviations

AIDS: acquired immune deficiency syndrome
CSM: common sense model
DAAs: direct acting antivirals
HCV: hepatitis C virus
HIV: human immunodeficiency virus
PWID: people who inject drugs
SVR: sustained virologic response
